# Some Practical Considerations for Compression Failure Characterization of Open-Cell Polyurethane Foams Using Digital Image Correlation

**DOI:** 10.3390/s20154141

**Published:** 2020-07-25

**Authors:** Ricardo Belda, Raquel Megías, Norberto Feito, Ana Vercher-Martínez, Eugenio Giner

**Affiliations:** 1 Centre of Research in Mechanical Engineering—CIIM, Department of Mechanical Engineering and Materials, Universitat Politècnica de València, Camino de Vera, 46022 Valencia, Spain; norfeisa@upvnet.upv.es (N.F.); eginerm@mcm.upv.es (E.G.); 2 Institute of Mechanical and Biomechanical Engineering—I2MB, Universitat Politècnica de València, Camino de Vera, 46022 Valencia, Spain; ramedia@upv.es (R.M.); anvermar@dimm.upv.es (A.V.-M.); 3 Networking Biomedical Research Centre in Bioengineering, Biomaterials and Nanomedicine (CIBER-BBN), Universitat Politècnica de València, Building 9C, Camino de Vera s/n, 46022 Valencia, Spain

**Keywords:** digital image correlation, compression fracture, speckle quality, failure characterization

## Abstract

(1) Background: Open-cell polyurethane foam mechanical behavior is highly influenced by microstructure. The determination of the failure mechanisms and the characterization of the deformation modes involved at the micro scale is relevant for accurate failure modeling. (2) Methods: We use digital image correlation (DIC) to investigate strain fields of open-cell polyurethane foams of three different densities submitted to compression testing. We analyze the effect of some DIC parameters on the failure pattern definition and the equivalent strain magnification at the apparent ultimate point. Moreover, we explore speckle versus non-speckle approaches and discuss the importance of determining the pattern quality to perform the displacement correlation. (3) Results: DIC accurately characterizes the failure patterns. A variation in the subset size has a relevant effect on the strain magnification values. Step size effect magnitude depends on the subset size. The pattern matching criterion presented little influence (3.5%) on the strain magnification. (4) Conclusion: The current work provides a comprehensive analysis of the influence of some DIC parameters on compression failure characterization of foamed structures. It highlights that changes of subset and step sizes have a significant effect on the failure pattern definition and the strain magnification values, while the pattern matching criterion and the use of speckle have a minor influence on the results. Moreover, this work stands out that the determination of the pattern quality has a major importance for texture analysis. The in-depth, detailed study carried out with samples of three different apparent densities is a useful guide for DIC users as regards texture correlation and foamed structures.

## 1. Introduction

Open-cell rigid polyurethane (PUR) foam is considered a reliable cancellous bone surrogate because of its similarities with regards to morphometry and mechanical behavior [1,2,3,4,5]. Its increasing relevance in the biomechanical field has motivated several investigations aiming at characterizing this foamed structures, either commercial [1,2,3,4,6,7] or self-produced [5,6]. This material presents some advantages with respect to real bone specimens, such as it does not suffer from dehydration or biodegradation and has a lower cost [1]. Hence, several works in the literature have used open-cell PUR foams to mimic healthy or osteoporotic cancellous bone, for the evaluation of orthopedic implants or cement augmentation [3,5,7].

Under quasi-static compression conditions, open-cell polyurethane foams response presents the following stages: linear elastic, softening after yield, plastic plateau and densification (at a high nominal strain) [8,9]. The mechanical behavior characterization of this kind of structures has been addressed from simplified [8,10,11] and realistic morphologies [3,4,5,6,12]. The simplified models developed by Gibson et al. considered the deformation modes involved (bending, elastic buckling and plastic collapse of the struts) as function geometrical features such as strut length and thickness and the apparent density [8,10,11]. Therefore, the determination and characterization of the deformation modes in open-cell foams is necessary to a more accurate mechanical behavior assessment, which is useful for many biomedical and engineering applications.

Advanced imaging systems such as SEM or micro-CT permit analysis of the microarchitectural characteristics of foam specimens [1,3,4,5,6]. Other works apply micro-CT during specimen testing to evaluate the deformation mechanisms of failure at different length scales [3,6]. The images resulting from this procedure may be analyzed using digital volume correlation (DVC) technique to estimate 3D displacement fields [13]. Moreover, image-based numerical models can be developed from the scans to simulate foam mechanical behavior with a high degree of discretization and assess the influence of morphometry [3,4,5,6,12]. However, the application of these techniques requires high economic and time costs.

The estimation of displacements in non-homogeneous or biological materials has a major importance to give insight into their failure mechanisms. Some of the most common measurement systems are loading platen transducers, displacement gauges, extensometers attached to specimen or optical systems [14]. Contact measurement systems may induce damage in the specimen, while loading platen transducers or displacement gauges provide apparent metrics. Among optical systems, digital image correlation (DIC) overcomes the cons mentioned and presents other advantages. For example, it provides both apparent and local metrics, which allows the detection of displacement field inhomogeneities at specimen surface. Moreover, in case of textured or heterogeneous materials, DIC technique uses microstructure as the grid of reference patterns to assess displacement fields [15,16].

Since DIC technique was proposed in the 80s [17,18], several investigations have applied it to measure displacement fields in a wide range of materials. At its first stages, it required time-consuming processing, which stimulated tracking algorithms development and improved its accuracy [19,20,21]. Nonetheless, DIC is limited to surface displacement measurement, so it only detects failures observed by the camera. In-depth surfaces failure cannot be distinguished although they affect to some degree the displacement estimation [15]. Its high potential for heterogeneous materials or biomechanical applications [22], from macro level failure characterization [23,24,25], to micro scale strain field estimation [26,27,28] has motivated several investigations.

Presently, local (or subset-based DIC) [17,18,21] and global (or FE-based DIC) approaches [29] are being investigated. Subset-based DIC processes each facet displacements independently and it is computationally more efficient, while FE-based DIC computes the displacement of all nodes simultaneously and can be linked to FE analysis [29]. Subset-based DIC is the most used approach. Several parameters have been identified as error sources, such as correlation criterion, shape function, subset size, step size, image noise, camera lens distortion or speckle quality [30,31,32,33].

The accuracy of subset-based DIC can be affected by many factors, and the user must rely on experience and intuition to set them up [30]. The selection of DIC parameters depends on the application but some general guidelines have been reported through the evaluation of real experiments or artificial patterns [30,31,32,34,35]. One of the most important DIC parameters is the subset size. Several works claim that a larger subset size reduces the displacement measurement error [30,33,36]. However, in those cases of non-homogeneous deformations, the accuracy of the strain field computation is reduced because the shape functions cannot describe the displacement gradients [30,36]. In case of small subset sizes, linear or quadratic subset shape functions can characterize displacement gradients. Random noise influence defines an inferior limit for the subset size [36].

DIC usually needs a speckle to track displacements, although some works have evaluated non-speckle correlation (also known as texture correlation) by using microstructural morphology as correlation pattern [15,26]. Speckle/texture characteristics have been proved to condition DIC accuracy. Therefore, speckle/texture pattern quality determination has attracted the attention of some investigations [30,34,35]. Parameters such as mean speckle size, subset entropy, sum of square subset intensity gradients (SSSIG), root mean square error (RMSE), mean intensity gradient or Shannon entropy have been applied to determine speckle quality [30,35,36].

On the other hand, some works have used DIC to characterize the heterogeneous strain field in PUR foams. Chiang and Ding [27] employed a multi-speckle technique (similar to DIC technique) to observe the differences in the strain field between macro and micro-size PUR samples when submitted to tensile loading. Although the deformation was almost uniform along the largest samples, the micro-size ones revealed heterogeneous patterns. Their multi-speckle technique results in lack of uniaxial failure initiation at each scale studied, which would have provided information about failure mechanisms. DIC has also been used to validate numerical models, as in Jin et al. [37], where 3D strain inhomogeneities during compression testing were observed and used the results to validate a viscoplastic foam model.

Other studies have used DIC to estimate fracture properties [28,33,38,39,40]. For example, Mokhtarishirazabad et al. [38] evaluated how DIC parameters affect the stress intensity factor estimation and found that not only subset size was important but also the size and position of the region of interest (ROI). On the other hand, local failure has been characterized using DIC [28,39,40]. For example, DIC allowed to assess the shear modulus through the visualization of the strain and displacement fields and to obtain the shear angle θ based on the principal strains [28], while the strain rate effect on the local failure was evaluated in [39].

The main goal of this work is to propose some guidelines to enhance DIC technique application to open-cell PUR foams compression characterization through displacement/strain fields assessment. A detailed analysis is performed about the influence of some DIC parameters (subset size, step size, pattern matching criterion, correlation criterion and use of speckle) on failure pattern localization and strain magnification estimation in the regions of failure. Moreover, the use of foam microstructure as correlation pattern (usually called texture correlation) is discussed and the speckle/texture quality is assessed through image Shannon entropy estimation.

## 2. Materials and Methods

### 2.1. Open-Cell Foam Specimens

**Figure 1:**
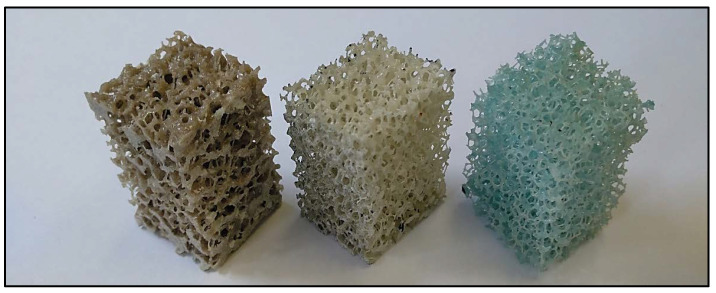
Specimens of different apparent densities analyzed in this work: High-density foam (HD) (**left**), medium-density foam (MD) (**center**) and low-density foam (LD) (**right**).

In this work, we analyze open-cell polyurethane foams of three apparent densities (Sawbones, Sweden) [41], Figure 1. The three apparent density grades are designed as follows: low-density foam (LD, Ref. #1522-507), medium-density foam (MD, Ref. #1522-524) and high-density foam (HD, Ref. #1522-525) [41]. For each foam grade, some foam properties are provided by the manufacturer, such as the apparent density ($\rho_{\mathrm{app}}$), the apparent compressive stiffness ($E_{\mathrm{app}}$), the compressive strength ($\sigma_{f}$) and foam volume fraction (FV/TV), summarized in Table 1. Furthermore, the manufacturer reports a mean pore size between 1.5 and 2.5 mm.

A series of 24 specimens, 8 of each density, were prepared from the 13 × 18 × 4 cm initial block. The parallelepiped-shaped specimens of 25 mm base side and 40 mm height average dimensions (see Figure 1) were machined using a table saw at low advance velocity and constant water irrigation. Furthermore, special attention was paid to get parallel faces at each specimen to avoid stress raisers during mechanical testing [14].

### 2.2. Compression Testing and Set up Definition

The specimens were tested under quasi-static compression conditions, under displacement control mode at a displacement rate of 1 mm/min. Tests were carried out using an electromechanical testing machine (MTS Criterion C42), with aluminum compression platens (MTS ref.: FYA502A) for the compression tests. The displacement between compression platens was measured using a displacement gauge (MTS ref.:632.06H-20) fixed to the upper plate. A 10 N preload was defined and the load-displacement response was recorded at a 10 Hz data acquisition rate.

**Figure 2:**
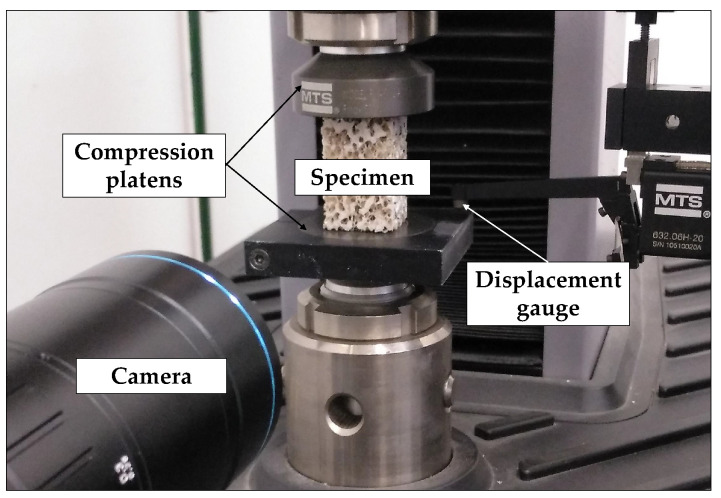
Set up used for open-cell polyurethane foams compression testing. A displacement gauge is used to measure the relative displacement of compression platens avoiding any compliance effect. A camera is positioned perpendicular to the specimen to acquire images during testing.

Moreover, a camera with a high resolution fixed focal lens (HF7518V-2, Myutron, Japan) and extension rings of 10 mm (focal length of 65 mm) was positioned perpendicular to the specimen to register images during testing at a 2 Hz image acquisition rate. Specimen-camera perpendicularity was manually adjusted and has a major importance to avoid out-of-plane effects that could affect DIC displacement field estimation.The dimensions of the field of view were 57.12 × 47.78 mm (2447 × 2047 pixels) and the pixel dimension is approximately 0.02 mm side. Moreover, we performed a lens distortion calibration [42]. The imaging system also includes a spotlight. The images were analyzed after testing through the application of DIC technique using VIC-2D Digital Image Correlation software (v.6.0.2 Correlated Solutions Inc., Irmo, SC, USA). The test set up is shown in Figure 2.

### 2.3. Digital Image Correlation Technique

**Figure 3:**
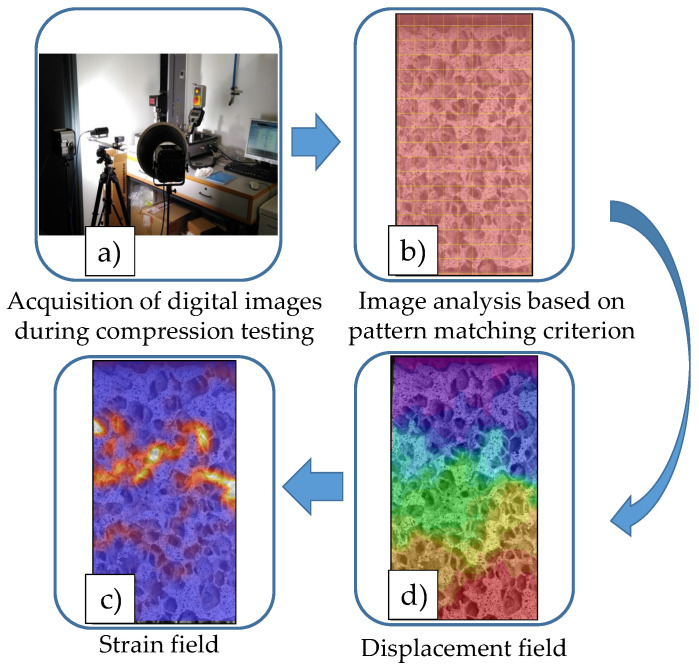
Scheme of digital image correlation (DIC) procedure. DIC is applied to (**a**) the images acquired during testing, which (**b**) are analyzed based on a pattern matching criterion, resulting in (**c**) the displacement field estimation. (**d**) The strain field is computed according to the chosen tensorial description.

This optical technique permits assessment of displacements and strains from the analysis of images taken during testing. A pattern matching criterion is used to calculate the displacement field from which the strain field is derived, see Figure 3.

The subset-based DIC method divides the region of interest into squared faces to track their displacement. To determine them, it requires an image matching method, which minimizes the difference between the reference (*F*) and deformed (*G*) images gray values. Assuming no lighting changes between images, the facet motion is determined using the sum of squares deviation (SSD), Equation (1) [21]. To further deformation estimation, the cost function needs to include a subset shape function to transform the coordinates after deformation [21].

(1) $$\chi_{\mathrm{SSD}}^{2}=\sum {\left(G_{i}-F_{i}\right)}^{2}$$

However, the SSD criterion assumes no changes in lighting between images, which is far from real experiments. To solve this issue, a photometric function (Φ) is applied to the gray values of G [21]. If the photometric transformation consists of a scaling, then Φ(G)=aG and the cost function is $\chi^{2}=\sum {\left(aG_{i}-F_{i}\right)}^{2}$. The optimal value for the scaling factor ($a=a_{\mathrm{opt}}$), calculated through the cost function derivative, is $a_{\mathrm{opt}}=\frac{\sum F_{i}G_{i}}{\sum G_{i}^{2}}$. This leads to the normalized sum of squared difference criterion (NSSD), Equation (2) [21].

(2) $$\chi_{NSSD}^{2}=\sum {\left(\frac{\sum F_{i}G_{i}}{\sum G_{i}^{2}}G_{i}-F_{i}\right)}^{2}$$

In case of considering the combination of a scaling and offset, the photometric transformation is Φ(G)=aG+b. The cost function is $\chi^{2}=\sum {\left(aG_{i}+b-F_{i}\right)}^{2}$ and, after some algebra, considering $\bar{F}$ and $\bar{G}$ as the mean intensity values of the deformed and reference images and ${\bar{F}}_{i}=F_{i}-\bar{F}$ and ${\bar{G}}_{i}=G_{i}-\bar{G}$, the expressions for the optimal values for the scaling ($a_{\mathrm{opt}}$) and offset ($a_{\mathrm{opt}}$) are $a_{\mathrm{opt}}=\frac{\sum {\bar{F}}_{i}{\bar{G}}_{i}}{\sum {\bar{G}}_{i}^{2}}$ and $b_{\mathrm{opt}}=\bar{F}-\bar{G}\frac{\sum {\bar{F}}_{i}{\bar{G}}_{i}}{\sum {\bar{G}}_{i}^{2}}$. This leads to the zero-mean normalized sum of squared difference (ZNSSD), Equation (3) [21]. Further details of the subset-based template matching method can be found in [21].

(3) $$\chi_{\mathrm{ZNSSD}}^{2}=\sum {\left(\left(\frac{\sum {\bar{F}}_{i}{\bar{G}}_{i}}{\sum {\bar{G}}_{i}^{2}}G_{i}-\bar{G}\frac{\sum {\bar{F}}_{i}{\bar{G}}_{i}}{\sum {\bar{G}}_{i}^{2}}\right)-\left(F_{i}-\bar{F}\right)\right)}^{2}$$

#### DIC Parametric Study

DIC technique has some internal parameters that may influence the displacement fields and strain fields estimation. Therefore, we aim at finding the parameters that have a greater effect on DIC solution and quantify their effect regarding failure pattern definition and strain magnification at failure. Hence, subsequently, we analyze changes in facet size, step size, pattern matching criterion, incremental correlation and speckle. The DIC parameter variation is studied on the same images for several specimens. In the case of the use of speckle, its effect is discussed through the analysis of strain fields and failure pattern comparison between DIC prediction and visual inspection of the tested samples.

**Figure 4:**
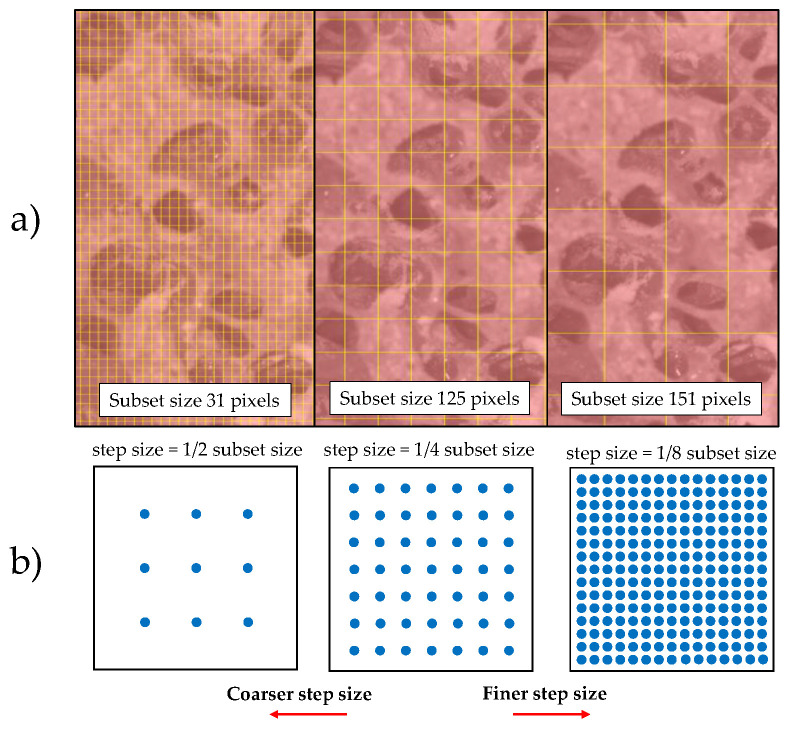
Representation of (**a**) step and (**b**) subset sizes variation.

The facet (or subset) size controls the area of the grid that is used to track the displacement between images, see Figure 4 top. It must ensure that there is sufficient pattern inside the area to perform the correlation [21,42]. Step size controls the spacing of the points analyzed during correlation, see Figure 4 bottom, and is recommended to be roughly 1/4 of the subset size, although the smaller the step size is, the more accurate displacement estimation [42]. On the other hand, the criterion used for pattern matching and the reference image used for the correlation (incremental or non-incremental correlation) may have influence on the strain field determination. In case of the use of speckle, homogeneous materials need for a speckle that acts as a pattern for displacement correlation. However, for non-homogeneous materials, microstructure may be enough.

Specifically, we study the following parameters/option variation:

- Facet (or subset) size: 31, 51, 81, 101, 125, 151 pixels size.
- Step size: 5, 10, 15, 20 pixels size.
- Pattern matching criterion: SSD, NSSD, ZNSSD.
- Incremental vs. non-incremental correlation
- Speckle vs. non-speckle

**Figure 5:**
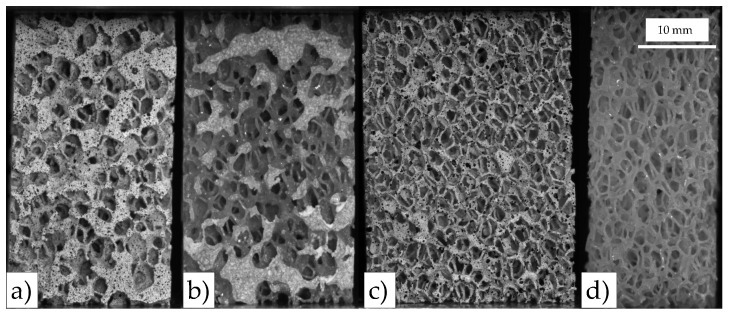
Speckled and non-speckled specimens example: (**a**) HD6 speckled, (**b**) HD8 non-speckled, (**c**) LD6 speckled and (**d**) LD7 non-speckled.

Speckle was applied to the visible surface of half of the specimens. It needs to fulfil some requirements, such as random distribution, non-repetitive, high contrast and its size depends on the application [21,42]. First, a white paint was used to increase contrast and then a black spray paint was applied to ensure speckle randomness. Speckled and non-speckled specimens are shown in Figure 5.

The quality of a speckle can be determined from image Shannon entropy (H) estimation [34]. The highest H is the lowest the estimation errors. We have calculated Shannon entropy for each ROI analyzed, speckled and non-speckled, following Equation (4).

(4) $$H=-\sum_{j}p\left(a_{j}\right)log\left(p\left(a_{j}\right)\right)$$

where *j* represents each gray level and $p(a_{j})$ is the normalized probability of gray level occurrence, which can be computed using the image histogram [34]. This enables the determination of the quality of the speckle or the texture pattern used to perform the displacement correlation and explores differences as a function of the foam apparent density.

One-way analysis of variance (ANOVA) was performed to compare means between groups using Statgraphics Centurion XVII (StatPoint Technologies, Inc., Warrenton, VA, USA). The level of significance was set to 5% (*p*-value < 0.05).

## 3. Results

### 3.1. Description of Displacement Field during Compression Testing

**Figure 6:**
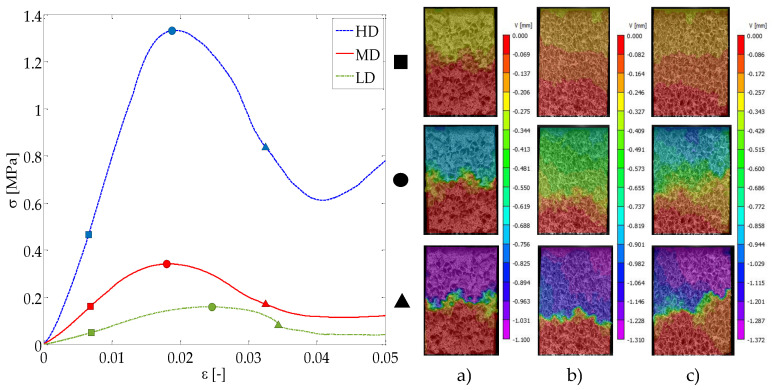
Stress-strain compression response registered for three specimens of different apparent density (**left**). Displacement field for (**a**) HD, (**b**) MD and (**c**) LD specimens (**right**), at the linear stage (square marker), ultimate point (circle marker) and post-yielding (triangle marker).

The compression response of open-cell polyurethane foams can be divided into a linear stage where the cells deform reversibly, a yielding region up to the ultimate point and a post-yielding non-linear region characterized by a softening region followed by a densification stage at high nominal strains, shown in Figure 6 for three samples of different apparent densities. The higher the apparent density is, the greater the apparent stiffness and the ultimate strength. In case of the ultimate strain, the samples of lower density (LD group) suffer from greater deformations prior to failure, see Figure 6 left.

Figure 6 (right) shows the non-homogeneous displacement field during compression testing for the three foam density groups. The displacement heterogeneities observed result from the foam microarchitectural characteristics of each specimen. As the nominal strain increases, the non-homogeneous displacements tend to stack in failure bands, notice the displacement transition from the linear response (square markers in Figure 6) to the post-yielding state (triangle markers in Figure 6). As failure evolves, the rest of the foam cells reduce their deformation and the displacement field becomes more homogeneous far from the failure region. Compression failure in foamed structures is more localized rather than spread and it is highly influenced by microstructure. The bi-dimensional failure patterns observed correspond to the failure of several struts. Some inclined fracture planes detected by DIC are maximum shear planes at 45° with respect to the applied compressive load, see Figure 6c) bottom.

Moreover, we observed that the lower the apparent density is, the higher the nominal strain prior failure. Therefore, the cell struts bending is greater in the case of LD specimens. The heterogeneous distribution of struts in the open-cell foams makes that local failure occurs mainly due to strut bending and shear at the strut joints.

The DIC technique provides accurate displacement fields for failure detection and failure pattern localization. However, its bi-dimensional application is slightly influenced by the deformation of the in-depth planes [15,26]. DIC expects a homogeneous planar surface so its 2D application to foamed structures performs a bi-dimensional projection of the deformation mechanisms involved in the visible surface. Therefore, the 3D deformation mechanisms of the whole specimen cannot be captured using this technique.

Our application of DIC to compression failure characterization can only distinguish the failure pattern at the apparent level, but not local failure at the strut level. Therefore, it could be interesting to apply this procedure to a foam slice of a few millimeters depth to capture local failure. This may allow the estimation of failure strains at the strut level, avoiding the undesirable homogenization performed by DIC in whole specimen testing, because we detected that failure tends to be highly localized.

### 3.2. Influence of DIC Parameters on Failure Pattern and Strain Values Estimation

With regards to the parametric study, for subset size, step size, correlation criterion and considering incremental or non-incremental correlation, we analyzed 2 samples, speckled and non-speckled, of each density grade and we refer the results to the equivalent strain magnification, defined as the ratio between the maximum equivalent strain at fracture at local level and apparent strain measured at the apparent ultimate point, ($\varepsilon_{\mathrm{mag}}=\frac{max(\varepsilon_{\mathrm{eq},f})}{\varepsilon_{\mathrm{app},u}}$). On the other hand, the effect of using speckle was assessed through the analysis of the whole data set. Half of the specimens were speckled.

Our criterion for choosing appropriate parameters is based on failure pattern detection accuracy and absence of voids in the solution. Some parameters combination led to accurate results for our criterion, so we will refer the failure properties estimation to a set of DIC parameters.

#### 3.2.1. Subset Size

**Figure 7:**
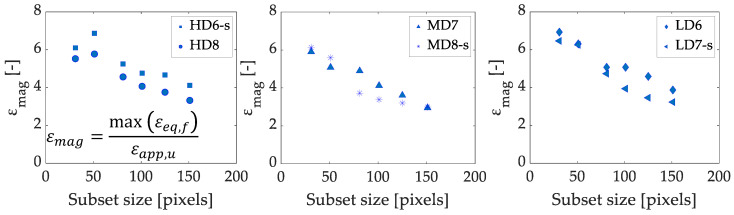
Subset size variation influence on the equivalent strain magnification at the apparent ultimate point for HD (**left**), MD (**middle**) and LD specimens (**right**). The extension -s denotes speckled specimens.

We analyzed the influence of the subset size for 31, 51, 81, 101, 125, and 151 pixels subset sizes, which correspond to between approximately 0.72 and 3.52 mm. In terms of the specimen height (*h*), which is the same for all the samples, the range of subset sizes is defined between 0.018 *h* and 0.088 *h*. In another investigation of our research group, we have characterized the morphometry of the foam groups analyzed in this work using micro-computed tomography [12]. A mean pore size of 2.36 mm, 2.16 mm and 2.45 mm was estimated for the HD, MD and LD groups respectively [12]. Therefore, the subset sizes analyzed are in the range between approximately 0.3 and 1.5-times the mean pore size. The results for the strain magnification ($\varepsilon_{\mathrm{mag}}$) for each foam grade, speckled and non-speckled, are shown in Figure 7. Higher strain magnifications were registered for the lowest density for the same combination of DIC parameters. The tendency observed is that the larger the subset size is, the lower the strain magnification at failure, and the values tend to converge for increasing subset size. A variation of the subset size between 51 and 151 pixels changes the strain magnification a 74% for the HD group. In case of MD and LD specimens, differences of 100% were found by changing the subset size between 31 and 151 pixels.

The differences observed between speckle and non-speckle approaches are not equivalent between foam grades, for example, the speckled sample HD6 has greater strain values, while LD7 speckled sample has lower strain values than the non-speckle LD specimen. Therefore, these differences may result from specimen variability rather than because of the speckle or non-speckle approach.

In the literature, several works investigate the effect of the subset size on displacement or strain measurement [30,31,32,33,36]. Some of them use artificial deformation fields to evaluate DIC performance [31,32]. Different behavior has been reported according to the strain amplitude or the heterogeneities in the strain field [30,32,33,36]. For example, Yaofeng and Pang [36] studied uniform and non-uniform deformations and stated that for the latter case, a mismatch of displacement functions and true deformation may occur due to high strain gradients. On the other hand, Bornert et al. [32] observed that for large strains, there is an increase of the global error with the subset size, which results from the lack of capability of the affine shape function to describe the local displacements. They report the opposite trend for small strains.

Lucas–Kanade displacement tracking algorithm assumes small deformations inside the subset. However, failure involves important displacement gradients, which are not well captured in large subset sizes. This has been observed in other works which claim that for large displacement gradients and large subset size, a first order shape function cannot properly describe the true deformations [30,32,33,36]. Therefore, the subset size needs to be large enough to contain sufficient pattern to perform the correlation but small enough to be able to capture the displacement gradients using first or second order subset shape functions. For large deformation analysis, a small subset size would be recommended, to describe the strain gradients properly [32].

**Figure 8:**
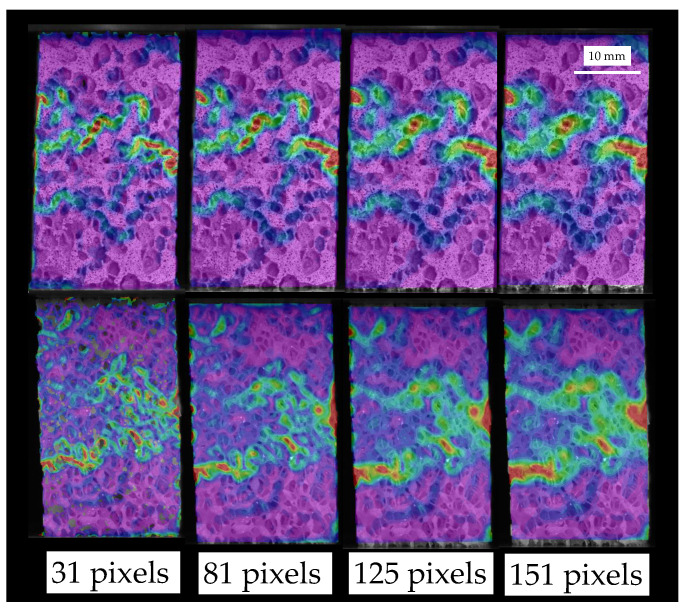
Subset size variation influence on the maximum equivalent strain measured in the failure region for HD (**top**), MD (**bottom**). The influence for LD specimens is very similar to the one depicted for the MD specimen.

Failure is a local phenomenon, so subset size should be large enough to permit DIC to solve the correspondence problem but small enough to represent the local effect. Small subset sizes may lead to voids on the solution, while large subsets may homogenize the solution, describing the whole specimen failure rather than the individual strut one, as depicted in Figure 8. Therefore, taking into account the mean pore size of each foam group (2.36 mm for HD, 2.16 mm for MD and 2.45 mm for LD), we propose as the best subset size the smallest one possible which permits the solution of the correspondence without producing voids in the displacement field estimation. Specifically, a subset of 81 pixels (0.8-fold the mean pore size) is recommended. This highlights that for texture correlation of foamed materials a subset size of the order of the pore size should be chosen.

#### 3.2.2. Step Size

**Figure 9:**
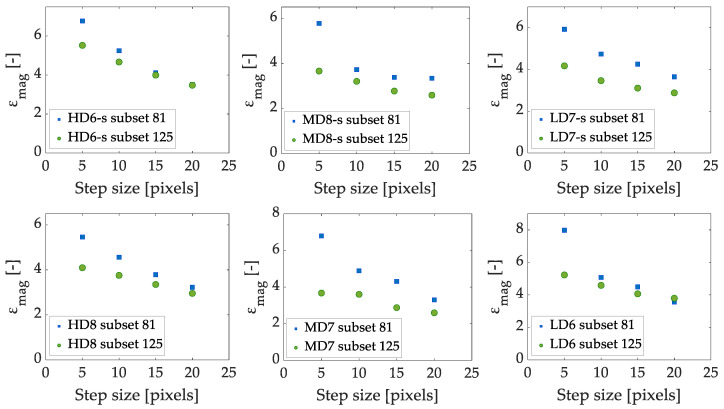
Effect of step size variation on the equivalent strain magnification at the apparent ultimate point for speckled specimens HD6, MD8 and LD7 (**top**) and non-speckled specimens HD8, MD7 and LD6 (**bottom**) for 81 and 125 pixels subset size. The extension -s denotes speckled specimens.

Step size parameter controls the distance between the points over which the correlation is made. Therefore, a low number is recommended to increase accuracy [42]. We vary the step size between 5 and 20 pixels, exploring its effect for 81- and 125-pixel subset sizes (values arbitrarily chosen in the range of subset sizes studied). Figure 9 shows the effect of step size variation on the equivalent strain magnification at fracture for HD, MD and LD speckled and non-speckled specimens. A clear influence is observed, a higher step size produces a lower equivalent strain magnification at fracture with a difference greater for lower subset sizes. The trend observed is independent of the use of speckle, see Figure 9.

Our results reveal that step size affects differently according to the subset size chosen, see Figure 9. For example, a maximum difference of 93% in the equivalent strain magnification is observed for the HD specimen for a subset size of 81 pixels. A similar influence is found for MD (100%) and LD (124%) specimens for the same subset size. On the other hand, a lower influence of the step size is observed for a subset size of 125 pixels. A maximum difference of 59% was found for HD, 41% for MD and 45% for LD specimens, see Figure 9.

**Figure 10:**
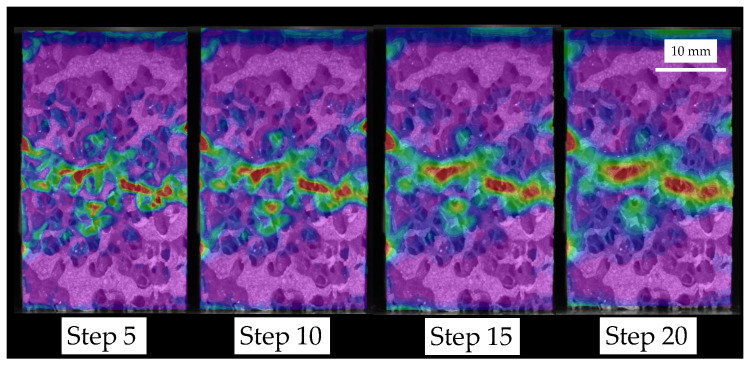
Influence of step size variation between 5 and 20 pixels on the equivalent strain field for a HD specimen. A similar influence was observed for MD and LD specimens.

The step size variation influence on the equivalent strain field is depicted in Figure 10 for a HD specimen. Increasing the step size, i.e., the distance between points to perform the correlation, tends to spread failure localization, but reduces computation time. Therefore, a larger step size homogenizes the strain distribution at the failure region. The step sizes analyzed describe with a good level of accuracy the experimental fracture pattern. Then, we choose a step size of 5 pixels, which corresponds to approximately 0.1 mm, because it has proved to provide an accurate compression failure description at a feasible computation time.

Summarizing, regarding the step size, we observed that the strain magnification decreases as the step size increases. We observed a loss of resolution in the failure pattern detection for an increase of the step size. This is a result of the reduced DIC ability to describe strain gradients for large step sizes.

#### 3.2.3. Correlation Criterion

**Figure 11:**
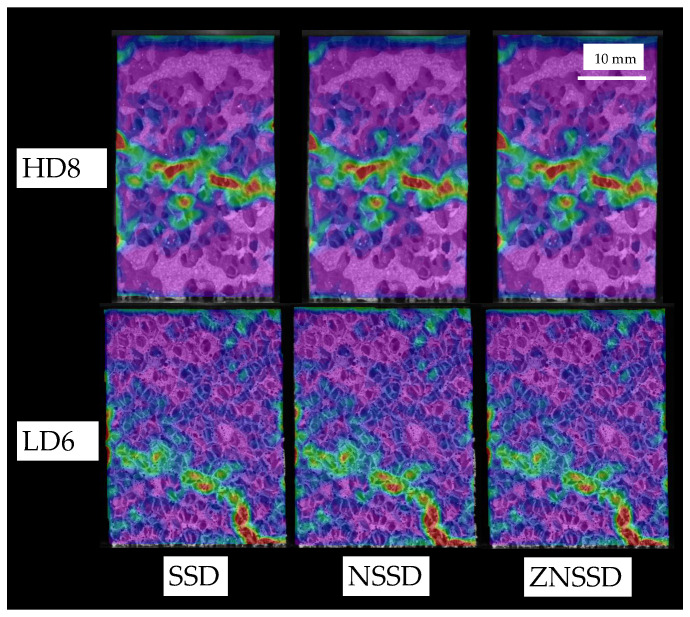
Equivalent strain distribution for a high-density (HD) specimen (**top**) and a low-density (LD) specimen (**bottom**), for sum of squared differences (SSD) (**left**), normalized squared differences (NSSD) (**middle**) and zero-normalized squared differences (ZNSSD) pattern matching criterion (**right**).

Choosing between squared differences (SSD), normalized squared differences (NSSD) or zero-normalized squared differences (ZNSSD) pattern matching criterion has a negligible influence on strain distribution results, as shown in Figure 11. No significant differences were found for both speckled (Figure 11 bottom) and non-speckled (Figure 11 top) approaches.

**Figure 12:**
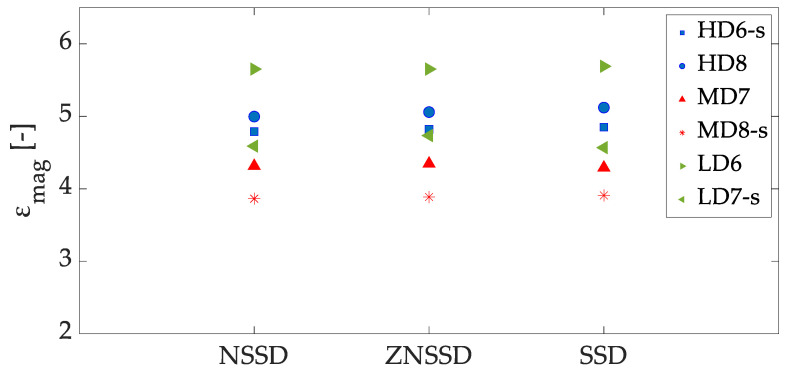
Representation of strain magnification at the apparent ultimate point as a function of the pattern matching criterion. Maximum differences of 3.5% were found between criterion. The extension -s denotes speckled specimens.

However, we found slight differences on the equivalent strain magnification values. In case of HD specimens, a 2.5% maximum difference was found due to pattern matching criterion. MD specimens presented a 1.3% difference in the strain magnification values. For the LD group, these differences increase up to a 3.5% between SSD and NSSD and ZNSSD criterion, Figure 12.

Nonetheless, we recommend the use of ZNSSD criterion because it assumes that changes in lighting may occur. This has been also stated in other works in the literature. For example, Wang et al. [29], reported some practical considerations for the use of DIC. They claim that displacement error is reduced through the use of a robust correlation criterion, such as ZNSSD, combined with a Gaussian pre-filtering of the image to reduce its noise [29].

#### 3.2.4. Incremental vs. Non-Incremental Correlation

**Figure 13:**
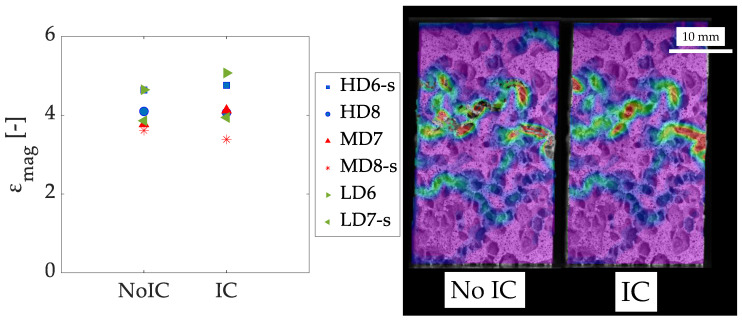
Equivalent strain magnification at the ultimate apparent point (**left**) and representation of the equivalent strain field (**right**) for incremental and non-incremental correlation.

Considering incremental correlation, i.e., comparing each deformed image to the previous one instead of the reference image is relevant. In both cases, the equivalent strain field does not differ much (Figure 13 (right)), but voids appear in the non-incremental correlation (No IC) case solution, so the correspondence problem is not properly solved.

This is explained by the significant deformations that occur at fracture, which DIC is not always able to capture in the comparison with the reference image. In Figure 13 (left), there are significant differences in the equivalent strain magnification values (between 5% and 12% in the cases analyzed). It can be noted that non-incremental correlation leads to lower strain magnification values, which can be explained because the largest strains are not detected (they correspond to the voids on the solution in Figure 13 (right) for the non-incremental correlation case). Anyway, the presence of voids on the strain field makes the non-incremental correlation to be discarded. This consideration has a great importance for large deformation situations, such as fracture characterization.

#### 3.2.5. Using Speckle in a Reticular Structure

DIC usually needs a speckle that acts as a pattern to perform displacement correlation between images. However, non-homogeneous materials, such as foams, can be analyzed using microstructure as the correlation pattern. To explore the quality of speckle and non-speckle approaches, we calculate the gray scale image Shannon entropy H [34] of each apparent density group.

Table 2 shows the mean and standard deviation values of image Shannon entropy H of each group. The mean H values of the speckled specimens are very similar for all the groups (7.49 for HD, 7.42 for MD and 7.38 for LD specimens). The ANOVA analysis revealed no significant differences between the speckled specimens (*p*-value = 0.12). In case of non-speckled specimens, the mean H values between foam groups are significantly different (*p*-value = 0.0025). On the other hand, the mean Shannon entropy values present larger differences between the three density foams analyzed. In HD speckled and non-speckled specimens, those differences are less significant (*p*-value = 0.045) than for MD (*p*-value = 5 × 10${}^{-6}$) and LD groups (*p*-value = 0.0028).

**Figure 14:**
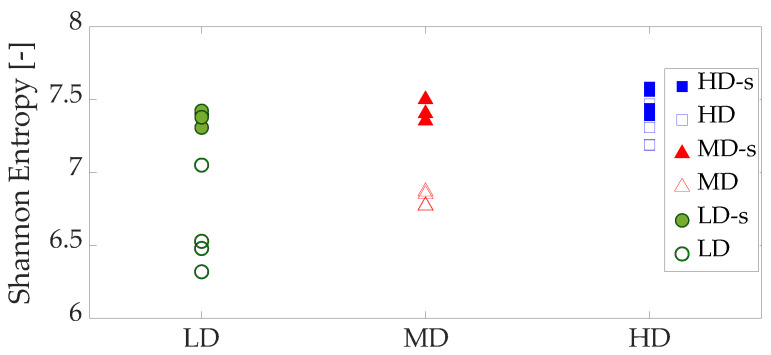
Image Shannon entropy results of each specimen analyzed, speckled (-s) and non-speckled.

Figure 14 depicts the Shannon entropy H values calculated for each specimen. The significant differences anticipated by the ANOVA analysis can be noted in the plot, which are less important for the HD group. The application of a speckle in a reticular structure increases the H values and produces patterns of low displacement errors and very similar entropy H values for the different apparent densities studied.

Table 3 summarizes the results of the equivalent strain magnification ($\varepsilon_{\mathrm{mag}}$) measured at the apparent ultimate point for each density grade and distinguishing between speckle and non-speckle groups. The mean value and standard deviation for each group are given. A mean value of the strain magnification for speckled HD foams of 6.74 was estimated, while we found a mean value of 6.33 for the non-speckled HD specimens. In case of MD specimens, a mean value of 3.54 was measured for speckle group and 4.22 for the non-speckle one. On the other hand, LD specimens also present little differences between speckled (4.16) and non-speckled (4.18) groups, Table 3. An ANOVA analysis has revealed no significant differences between the average values of speckled and non-speckled specimens (*p*-value > 0.05). Therefore, little differences were observed between speckle and non-speckle groups, with no clear dependence of the apparent density. Hence, those differences may be attributed to inter-specimen expected variability rather than the speckle/non-speckle approach. Nonetheless, the standard deviation values in the non-speckled specimens are larger than in the speckled ones, which may result from a decrease in the strain field estimation for non-speckled specimens.

**Figure 15:**
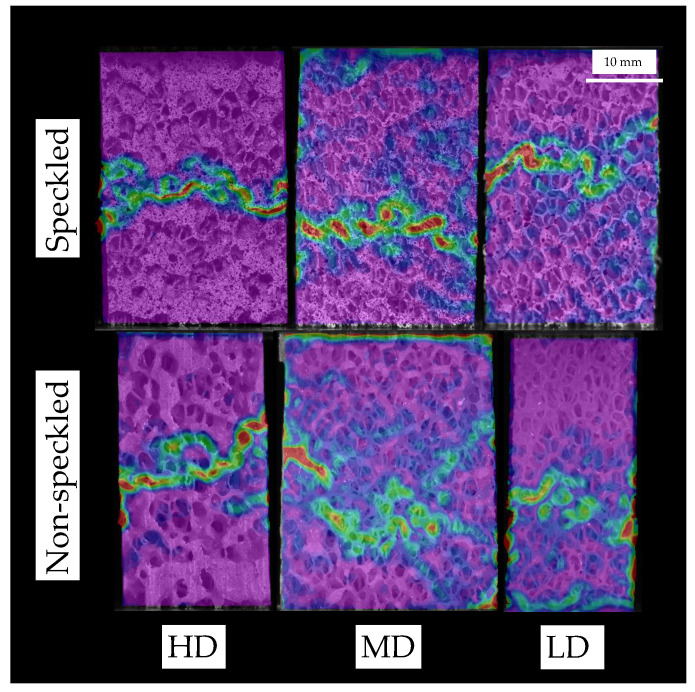
Equivalent strain distribution for speckled and non-speckled specimens. Slight differences are found in failure pattern determination. The failure region is more spread in the non-speckle than in the speckle approach.

Figure 15 depicts equivalent strain distribution for post-yield states of speckled (top) and non-speckled (bottom) specimens. Qualitatively, both approaches are equivalent regarding failure detection, but in case of non-speckled specimens it seems to be a bit more spread than for the speckle approach. This is related to a greater contrast achieved using speckle that enhances pattern matching and higher Shannon entropy H values associated with the speckled specimens. Based on our results, speckled and non-speckled approaches are equivalent regarding failure strains measurement and their performance is similar about failure detection, but the speckle approach localize more failure, which is slightly more spread in the non-speckle approach.

The results presented in this section reveal that the determination of the speckle/texture quality in foamed materials has a major importance to have accuracy on the displacement field estimation. Therefore, in case of texture correlation we recommend assessing the speckle/texture quality to choose whether it is necessary to apply a speckle to the material texture.

We found that speckled samples lead to very similar Shannon entropy values between foam groups. This points out that the application of a speckle to a textured material leads to a pattern of similar characteristics, despite the microstructural characteristics of each foam grade. The values obtained are very similar than the ones obtained by Liu et al. [34] for the lowest error in the estimation. Liu et al. [34] explored Shannon entropy H parameter to determine speckle quality and studied laser speckle, painted speckle and texture speckle. They concluded that the lowest displacement error was achieved for the highest Shannon entropy speckles, which corresponded to painted speckle [34].

In the literature, other approaches to determine speckle quality have been proposed. Yaofeng and Pang [36] introduced a new concept called subset entropy for carrying out an outstanding choice of the subset size. It consists of the sum of absolute difference of the intensities of the eight neighboring pixels. Pan et al. [30] proposed the Sum of Square of Subset Intensity Gradients (SSSIG) parameter to analyze speckle quality and to select subset size. They related this parameter to the subset entropy and stated that their methodology is mathematically supported, while subset entropy is based on an intuitive idea [30].

### 3.3. Strain Magnification at the Apparent Ultimate Point Strains Using DIC Results

The parametric DIC results give insight into the effect of DIC on the strain magnification values and on the failure pattern definition. There is not a unique combination of parameters that gives the best results, so there is a need to refer our results to a combination of parameters.

The selection of those parameters depends on the absence of voids on the solution, minimization of noise and localization of failure pattern. The subset size should be large enough to contain sufficient speckle/texture within it to solve the correspondence problem. Step size is recommended to be as low as possible to increase the solution accuracy. Incremental correlation, which compares each deformed image with the previous instead with the reference image, has a major importance to avoid holes on the solution, while a filter size large enough must be selected to avoid strain blurring that makes the fracture pattern detection vanish. Thus, the following settings were selected: subset size of 81 pixels, step size of 5 pixels, ZNSSD pattern matching criterion, incremental correlation and a filter size of 21 pixels.

**Figure 16:**
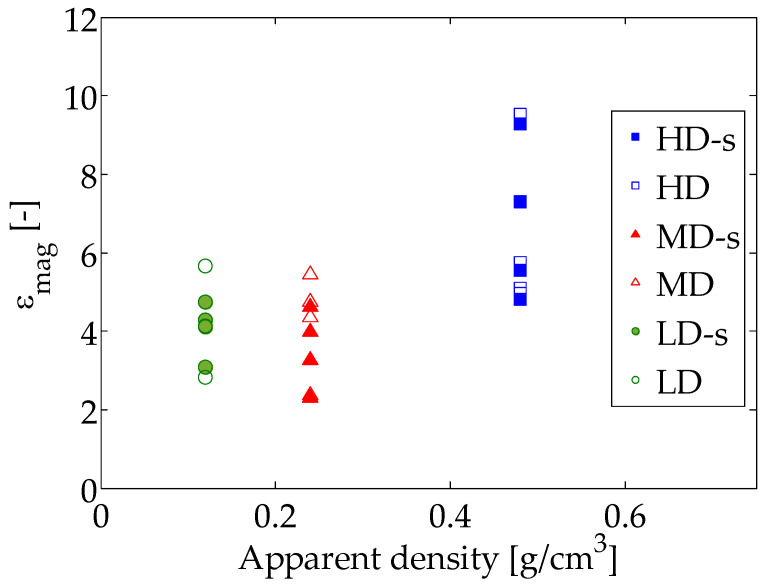
Scatter plot of the equivalent strain magnification at the apparent ultimate point as a function of the apparent density group of the samples.

The strain magnification values obtained for each specimen, speckled and non-speckled are depicted in Figure 16. Some variability is found in the results, which points out that the strain magnification amplitude of each foam specimen is influenced by its morphological characteristics. Table 4 shows the average values and standard deviation of the equivalent strain magnification at the apparent ultimate point. A mean value of 6.54 was found for the HD group. MD specimens presented a mean strain magnification value of 3.88, while LD specimens 4.12. The strain magnification of the HD group is significantly higher (*p*-value = 0.0003) than for MD and LD groups, see Figure 16. The differences in strain magnification values between groups can be influenced by the lowest apparent strain registered at failure for the HD group (mean value of 1.89%), while MD and LD specimens apparent strain at the ultimate point were very similar, 2.3% and 2.5% respectively.

We did not find any work in the literature that reports failure strains neither strain magnification measured using DIC for open-cell polyurethane foams. Koohbor et al. [39] evaluated differences on the local and global strain response at failure, but from a dynamic perspective. They found that the local strain rates were about an order of magnitude higher than the global ones [39], which would be an extension of our analysis for dynamic loading. Wang et al. [16] studied deformation patterns on polymeric foams using DIC, reporting material densification but they did not provide any comparison with experimental observations neither strain magnifications. However, contrary to our observations, they claim that high density foams show no localization of deformation at any apparent deformation. The lack of experimental observations to compare with in [16] reduce the validity of their DIC results. On the other hand, [43] also analyzed strain heterogeneities using DIC and they reported failure to be localized in fracture bands rather than spread, in line with our observations. Other studies used a combination of DIC, experimental tests, and finite element modeling to estimate failure parameters for complex loading simulation [40]. However, the DIC results in [40] presented voids in the solution, which points out that the DIC parameters used were not appropriate for the analysis.

## 4. Limitations

We acknowledge some limitations of the study. First, the DIC technique is conceived for a homogeneous planar surface, so we obviously detect the 3D displacement projection to the outer surface. However, we consider that its effect is minor in our study. We refer the results to the strain magnification, which assumes the aforementioned projection in its definition. Moreover, the displacement response is dominated by the compression direction, which is contained in DIC plane of analysis. Despite the 2D application, DIC performance is affected by the in-depth plane displacements, so failure pattern detection may be slightly influenced. However, it is considered that this fact has little influence on the displacement field estimation [15]. On the other hand, in this study we conduct a direct application of DIC to characterize strain fields at failure. The local displacement could not be compared with the metric from any other measurement system. Therefore, the study lacks an analysis of the absolute error committed. This is often investigated in the literature through the application or artificial known deformations as a reference. The use of artificial deformations overcomes the lack of an external local measurement to perform the comparison.

## 5. Conclusions

This work provides a comprehensive parametric analysis of the influence of a variation of some DIC parameters on the strain field estimation and the strain magnification at failure. Moreover, a texture correlation approach, which uses the foam microstructure as a pattern for the correlation, has been studied. The results presented in this work reveal the need to determine the texture quality to decide whether a speckle is necessary in foamed microstructures.

On the other hand, we report the influence of some DIC parameters on the failure pattern detection and the strain magnification at the apparent ultimate point for open-cell polyurethane foams of three different densities. DIC performance is affected by the setup and the selection of the parameters in the study. For example, the subset size has an important influence (up to 100% difference for a variation between 31- and 151-pixels in size) on the strain magnification assessment. To describe the gradients at the failure zone, a compromise is needed: subset needs to include enough pattern to perform the correlation, but it needs to be capable of describing the displacement gradients. For our setup, we propose a subset size of 81 pixels, which corresponds to approximately 0.8-fold the mean pore size.

On the other hand, an increase in the step size reduces the strain magnification estimation and makes the failure detection less localized (the strain gradients are less pronounced). However, failure localization is accurately performed despite a variation in this parameter.

Pattern matching criterion has little effect on the strain magnification values (a maximum differences of 3.5% was found between SSD and ZNSSD). Anyway, it would be recommended to use ZNSSD criterion, because it accounts for slight changes during image acquisition and has been proved to reduce the displacement error estimation. On the other hand, using an incremental correlation has been found as mandatory for compression failure characterization, because of the large deformations involved.

We have detected that in non-speckle approaches, it is important to evaluate the texture quality, for example, through image Shannon entropy H estimation. It permits evaluation of whether the texture is a valid pattern to perform accurate displacement field estimation. We observed that some textures led to H values as high as the speckled specimens. Moreover, the application of a speckle improved the Shannon entropy values, which were similar despite the initial texture features of the sample.

As regards the strain magnification values at the apparent ultimate point, we found no significant differences (*p*-value > 0.05) between speckle and non-speckle approaches for the three density groups. The strain magnification of the HD group (average value of 6.54) was significantly higher (*p*-value = 0.0003) than the one of MD (3.88) and LD (4.12) groups.

Finally, in this work it has been proved that several DIC parameters influence the strain field values assessment and their definition depends on the experimental setup and the texture characteristics of the samples. The detailed DIC parameter analysis performed is useful for experimental investigations of foamed materials such as cancellous bone surrogates or bone specimens and for implant design optimization using surrogates.

## Figures and Tables

**Table 1:** Mechanical and morphological properties provided by the manufacturer for each open-cell graded foam from [41].

Foam Grade	Density [$g/{\mathrm{cm}}^{3}$]	FV/TV [%]	$\sigma_{f}$ [MPa]	$E_{\mathrm{app}}$ [MPa]
LD	0.12	10.6	0.28	18.6
MD	0.24	15.4	0.67	53
HD	0.48	30.8	3.20	270

**Table 2:** Image Shannon entropy H mean and standard deviation values of each foam grade, speckled and non-speckled.

**H [-]**	**Speckle**	**Non-Speckle**
HD	7.49 ± 0.094	7.29 ± 0.132
MD	7.42 ± 0.075	6.83 ± 0.053
LD	7.38 ± 0.049	6.59 ± 0.318

**Table 3:** Mean and standard deviation values of the equivalent strain magnification ($\varepsilon_{\mathrm{mag}}$) at the apparent ultimate point for speckle and non-speckle groups and the three densities analyzed.

**$\varepsilon_{\mathrm{mag}}$ [-]**	**Speckled**	**Non-Speckled**
HD	6.74 ± 1.99	6.33 ± 2.15
MD	3.54 ± 0.99	4.22 ± 1.32
LD	4.16 ± 0.70	4.18 ± 1.16

**Table 4:** Equivalent strain magnification ($\varepsilon_{\mathrm{mag}}$) at the apparent ultimate point. The results are presented as the mean value and standard deviation for each foam density.

**Sample**	**$\varepsilon_{\mathrm{mag}}$ [-]**
HD	6.54 ± 1.94
MD	3.88 ± 1.14
LD	4.12 ± 0.89

## Abbreviations

The following abbreviations are used in this manuscript:

DIC	Digital image correlation
DVC	Digital volume correlation
PUR	Polyurethane
SEM	Scanning electron microscopy
SSD	Sum of squares deviation
NSSD	Normalized sum of square difference
ZNSSD	Zero-mean normalized sum of square difference
STD	Standard deviation
ROI	Region of interest
